# Neurobiological and Hormonal Mechanisms Regulating Women’s Sleep

**DOI:** 10.3389/fnins.2020.625397

**Published:** 2021-01-14

**Authors:** Alanna Dorsey, Luis de Lecea, Kimberly J. Jennings

**Affiliations:** Department of Psychiatry and Behavioral Sciences, School of Medicine, Stanford University, Stanford, CA, United States

**Keywords:** estrogen, progesterone, sex difference, arousal, ovarian hormones

## Abstract

Sleep is crucial for optimal well-being, and sex differences in sleep quality have significant implications for women’s health. We review the current literature on sex differences in sleep, such as differences in objective and subjective sleep measures and their relationship with aging. We then discuss the convincing evidence for the role of ovarian hormones in regulating female sleep, and survey how these hormones act on a multitude of brain regions and neurochemicals to impact sleep. Lastly, we identify several important areas in need of future research to narrow the knowledge gap and improve the health of women and other understudied populations.

## Introduction

Nearly all living organisms spend a fraction of their lives in a reversible, unconscious, coma-like state, some experiencing fantastical dreams reminiscent of schizophrenic symptoms. It is still unclear why exactly organisms sleep, but the necessity of sleep for health and well-being is certainly clear. Disrupted sleep negatively impacts cognition, risk of disease and obesity, and numerous indicators of health (Meng et al., 2013; Vgontzas et al., 2013; Irwin, 2015; Schmid et al., 2015; Smith and Mong, 2019). Sleep strengthens and consolidates memory (Arzi et al., 2012; Diekelmann et al., 2012), and sleep disruption impairs this learning process (Drummond and Brown, 2001; Gujar et al., 2010) as well as other cognitive processes (Johnson et al., 2006). Sleep-deprived patients have a weakened immune response to vaccinations and are more vulnerable to immune challenges (Spiegel et al., 2002; Lange et al., 2003; Cohen et al., 2009). Long-term sleep deprivation may even lead to death (Rechtschaffen et al., 1989). In women, sleep deprivation is associated with an elongated and more painful labor (Chang et al., 2010), as well as a decline in physical performance in older age (Goldman et al., 2007). The impacts of sleep deprivation are far-reaching, revealing the crucial role of sleep in cognition, health, and general well-being (Table 1).

**TABLE 1:** Sleep disruption has vast health consequences.

Consequence of cleep deprivation	Symptoms	References
**Impaired cognition**	Impairs strengthening and consolidation of memory	Drummond and Brown, 2001; Gujar et al., 2010
	Inattentiveness and other ADHD-like symptoms	Dahl, 1996; Pilcher and Huffcutt, 1996; Dahl and Lewin, 2002
	Psychiatric disorders and substance abuse in adolescents	Kirmil-Gray et al., 1984; Roberts et al., 2000; Johnson and Breslau, 2001
**Increased risk of disease**	Common cold and pneumonia	Cohen et al., 2009; Patel et al., 2012
	Cardiovascular disease	Mullington et al., 2009; Vgontzas et al., 2013; Azevedo Da Silva et al., 2014
	Hypertension	Gangwisch et al., 2010; Meng et al., 2013; Palagini et al., 2013
	Alzheimer’s	Lucey and Bateman, 2014; Peter-Derex et al., 2015; Smith and Mong, 2019
**Other health indicators**	Immune response to influenza A and hepatitis A vaccine decreased by over 50%	Spiegel et al., 2002; Lange et al., 2003
	Increased appetite and risk of obesity	Brondel et al., 2010; Buxton and Marcelli, 2010; Schmid et al., 2015
	Longer and more painful labor during pregnancy	Lee and Gay, 2004; Chang et al., 2010

*Sleep deprivation impairs cognitive processes, increases vulnerability for a myriad of diseases, and disrupts other vital health indicators.*

Worryingly, there is a clear sex bias in reported sleep disorders with significant implications for women’s health. Women are 41% more likely than men to experience insomnia, and this risk increases with age (Zhang and Wing, 2006). Women are also at twice the risk of restless leg syndrome (RLS) (Berger et al., 2004). Frequently, women are more likely than men to report difficulty maintaining sleep, feeling unrefreshed in the morning, and excessive daytime sleepiness (Lindberg et al., 1997). Obstructive sleep apnea, however, is more prevalent in men (Paul et al., 2008). We will note here that the term “women” in this review refers to cis women (see section “Discussion”).

Given the detrimental effects of sleep disruption on health, cognition, and well-being, this predisposition for women to experience sleep problems potentially contributes to sex-linked health disparities, such as the greater prevalence of Alzheimer’s disease in women than men (Mielke et al., 2014). Therefore, to improve health outcomes for women across the lifespan, it is critical to understand how sleep manifests during different phases of a woman’s life. Furthermore, researchers must continue efforts to identify the biological source(s) of sex differences in sleep and identify potential substrates amenable to clinical intervention. Many lines of converging evidence point to a critical role for ovarian sex hormones in mediating sex differences in sleep and in acutely regulating female sleep. The present review will survey the field’s knowledge on sex differences in sleep and discuss recent insights into the role of sex hormones in sleep.

## Sex Differences in Sleep Disturbances

### Subjective Measures and Sleep Disorders

Women frequently report subjectively poorer sleep than men. Women are more likely than men to report difficulty staying asleep, feeling unrefreshed in the morning, and excessive daytime sleepiness (Lindberg et al., 1997). Women reported more disturbed sleep, such as more disturbance from noise (Rediehs et al., 1990). Women report longer sleep onset latency (the time from lights off to falling asleep) and more nocturnal awakenings (Mniszek, 1988; Janson et al., 1995; Li et al., 2002; Tsai and Li, 2004). These frequently reported symptoms converge such that a meta-analysis found that women are 41% more likely to experience insomnia than men (Zhang and Wing, 2006). Women are also more than twice as likely to experience anxiety and depressive disorders (Kessler et al., 2005; Bekker and Van Mens-Verhulst, 2007), which have been correlated with sleep disturbances (Paulsen and Shaver, 1991; Hohagen et al., 1993). However, the sex difference in sleep remained significant after controlling for psychological status, so the higher prevalence for anxiety and depression in women is not the sole reason for the observed sex difference (Lindberg et al., 1997). During puberty, post-menses girls have a 2.75-fold risk of insomnia compared to pre-menses girls while boys only have a slightly increased risk throughout the course of puberty, even after adjusting for comorbid psychiatric disorders. This sex difference only occurred after menses onset (Johnson et al., 2006), suggesting that the pubertal rise in gonadal hormones may play a causal role.

Women are also at higher risk for non-insomnia sleep problems. People with global sleep dissatisfaction (GSD) are more likely to report excessive daytime sleepiness and to use sleep medications. Like insomnia, GSD is more prevalent in women than men (Ohayon and Zulley, 2001). Women are also at twice the risk of experiencing RLS (Berger et al., 2004). RLS is characterized by an uncomfortable prickling sensation in the legs and the desire to move them, especially at night. Consequently, RLS causes difficulty falling asleep.

When considering the sex difference in reported sleep, it is difficult to distinguish between sex differences in perception of sleep quality and willingness to admit to symptoms. For example, women may report fewer symptoms of apnea because of the social awkwardness associated with women snoring. Therefore, it is possible that sex difference in diagnosis rates does not accurately reflect sex differences in sleep quality. However, it is generally accepted by most clinicians that women experience more sleep problems than men (Buboltz et al., 2001; Arber et al., 2009; van de Straat and Bracke, 2015).

### Objective Measures

Surprisingly, polysomnographic measures [including electroencephalography (EEG) and electromyography (EMG)] usually report women as having better objective sleep across age ranges. Young women fell asleep faster and had better sleep efficiency (the percentage of time spent asleep while in bed) than young men (Goel et al., 2005). Middle-aged women had better sleep efficiency, had more rapid-eye-movement (REM) sleep, and were better at maintaining REM sleep than men (Kobayashi et al., 1998). Women over the age of 58 had longer REM latencies (the time from sleep onset to the first appearance of REM sleep) than men of the same age (Rediehs et al., 1990). REM latency is a biomarker of sleep disorders, and longer REM latencies have been correlated with sleep apnea and periodic limb movements (Shrivastava et al., 2014). Women across multiple ages had more slow-wave sleep (SWS) than age-matched men (Hume et al., 1998; Fukuda et al., 1999). SWS is indicative of sleep regulation and intensity, with more SWS associated with more restful sleep. Sleep spindles are another indicator of sleep stability (Dang-Vu et al., 2010; Schabus et al., 2012) and could contribute to the better objective sleep experienced by women compared to men. Women have more sleep spindle activity than men both during adolescence (Goldstone et al., 2019; Markovic et al., 2020) and adulthood (Gaillard and Blois, 1981; Huupponen et al., 2002; Purcell et al., 2017). Therefore, by EEG measures and other polysomnographic indicators, women should experience *better* sleep. The mismatch between so-called objective and subjective sleep measures points to a more nuanced relationship between sex and sleep. These objective sleep measures may not be equally accurate in women as in the men they were largely designed for. Or, these differences in self-reported sleep could be due to a different willingness to seek medical help. Sleep itself could also function differently in men and women such that women truly experience poorer sleep despite increases in SWS and sleep efficiency. The reasons for this discrepancy will only be elucidated with increased attention to sex as a biological variable in future sleep research.

## Changes in Women’s Sleep During Key Life Transitions

There is considerable evidence that female sex hormones, namely, estrogens and progestogens, directly impact women’s sleep and thus likely mediate sex differences in sleep. Women’s sleep disturbances are most pronounced during periods of life characterized by significant hormonal change. Sex differences in subjective sleep arise in puberty, in which young women experience a surge of sex hormones and, concomitantly, are at almost three times the risk of developing insomnia relative to adolescent boys (Johnson et al., 2006). Some sex differences in objective sleep—such as sleep spindle activity—emerge prior to puberty, but even these differences are much more pronounced following menses (Markovic et al., 2020). Furthermore, women exhibit different sleep architectures during the different stages of their ovulatory cycle, highly correlated to their changing hormone levels (Moline et al., 2003; Lord et al., 2014). Women also report more sleep disturbances during periods of hormonal flux, including menses, pregnancy, and menopause (Mallampalli and Carter, 2014). During these periods, the absolute concentrations of and ratio between estrogens, progestogens, and androgens change dramatically. Furthermore, hormone fluctuations may also occur within a single hormone class. For example, the hormone class “estrogen” consists of four steroid hormones. Estradiol (E2) is the most prevalent type of estrogen for women in childbearing age and within most laboratory animal models. As a woman transitions through menopause, the most prevalent form of endogenous estrogen shifts from estradiol to estrone (E1). During pregnancy, estriol (E3) is the main estrogen produced by the ovaries, although estetrol (E4) is produced by the fetus at this time. Additionally, intentional changes to the hormonal profile, such as administration of exogenous hormone therapy or oral contraceptives (OCs), can also alter sleep (Baker et al., 2001a,b; Burdick et al., 2002). The following sections will review women’s sleep during each of these key phases with special focus on how women’s sex hormones correlate to changes in sleep.

### Menstrual Cycle

Most women experience regular cyclic changes in sex hormones lasting approximately 28 days, also known as the menstrual cycle. Ovarian hormone concentrations are regulated by the hypothalamic-pituitary-gonadal axis: neurons in the hypothalamus release gonadotropin releasing hormone (GnRH), which causes the anterior pituitary to release the gonadotropins luteinizing hormone (LH) and follicle-stimulating hormone (FSH) into the bloodstream, which in turn act on the ovary to regulate follicular development and the production of sex steroid hormones. The menstrual cycle can be separated into two phases. In the follicular phase (approximately days 1–14), an ovarian follicle matures and estradiol grows to peak in concentration. High estrogen permits LH and FSH to spike and trigger ovulation, while estradiol temporarily decreases. After ovulation, the ruptured follicle transforms into the corpus lutea and thus the luteal phase (approximately days 15–28) begins. LH and FSH decrease as estradiol slowly increases again and progesterone increases significantly. Estradiol, progesterone, LH, and FSH are at low concentrations as menses occurs and the cycle begins again.

Many studies have found changes in sleep architecture across the phases of the menstrual cycle, with most sleep disturbances occurring in the luteal phase. In the luteal phase, women experienced increased sleep onset and awakenings, and lower sleep efficiency and quality compared to the follicular phase (Manber and Bootzin, 1997; Baker and Driver, 2004). Women in the luteal phase had less REM sleep and more non-rapid-eye-movement (NREM) sleep, with an increase in SWS in particular (Schwierin et al., 1998; Baker et al., 2002). EEG power density varies throughout the menstrual cycle, with the highest density of sleep spindles occurring in the luteal phase (Driver et al., 1996). The luteal phase is also associated with elevated core body temperature, which could potentially interact with sleep processes to impact sleep quality (Baker et al., 2001a). During the luteal phase, some women experience premenstrual syndrome (PMS), which includes symptoms of discomfort such as stomachache, backache, headaches, and nausea. Those with PMS are especially vulnerable to sleep disruptions during the luteal phase. Women with PMS self-reported having more unpleasant dreams, nocturnal awakenings, morning tiredness, and increased mental activity at night in comparison with women without PMS (Mauri et al., 1988). Women with PMS are more likely to report insomnia and migraines premenstrually and are at greater risk for daytime sleepiness than non-symptomatic women (Sheldrake and Cormack, 1976; Manber and Bootzin, 1997). Considered together, these data indicate that women’s sleep varies across the menstrual cycle, with the most robust changes reported when both estrogen and progesterone concentrations are elevated.

There are also documented changes in sleep and sleep-related processes in cycling women who are taking OCs. Polysomnographic studies have found that women taking OCs, relative to naturally cycling women in the luteal phase, have less SWS but more light and REM sleep (Baker et al., 2001a,b; Burdick et al., 2002). Nocturnal body temperature is higher in women taking OCs than women in the follicular phase and young men, likely due to the thermoregulatory effects of synthetic progestins (Kattapong et al., 1995; Baker et al., 1998, 2001a,b; Stachenfeld et al., 2000; Burdick et al., 2002). Sleep has been associated with body temperature: onset of sleep provokes a decrease in core temperature (Barrett et al., 1993) and a rapid decline in core temperature promotes sleep initiation and deeper sleep (Murphy and Campbell, 1997). Sleep architecture during the menstrual cycle could be further changed due to synthetic steroids increasing core body temperature. Though many women take OCs for a significant portion of their lives, many questions remain regarding the impact of OCs on sleep.

### Pregnancy

Pregnancy is another period in which women experience dramatic changes in both sex hormone concentrations and sleep quality. The course of pregnancy is separated into three trimesters, with the first trimester corresponding to weeks 1–13 of gestation, the second trimester corresponding to weeks 14–26, and the final trimester corresponding to weeks 27–40. During pregnancy, there is a significant increase in maternal sex hormone production. Estrogen and progesterone concentrations increase as the fetus matures, and peak to about 50- or 60-fold (compared to non-pregnancy levels) shortly before delivery (Hardie et al., 1997). After parturition (childbirth), estrogen and progesterone rapidly decline and return to normal in 2 or 3 months postpartum. Likewise, prolactin increases linearly, up to sevenfold in late pregnancy, compared to early pregnancy (Rigg et al., 1977). Cortisol increases in the third trimester to twice the amount of non-pregnant women, and peaks to up to 4.7 times as high during labor (Allolio et al., 1990) before rapidly returning to normal following delivery.

Self-reported sleep disturbances increase in prevalence throughout the course of pregnancy; 68% of women report having altered sleep during pregnancy, which increases from 13% in the first trimester to 66% in the third trimester. The most commonly cited reasons for sleep disruption include urinary frequency, headaches, and leg cramps (Schweiger, 1972). In another survey, nearly all (97.3%) pregnant women report nocturnal awakenings, which increase in frequency and duration throughout pregnancy (Mindell and Jacobson, 2000). Also common were reports of restless sleep, difficulty falling and staying asleep, and daytime sleepiness, which increased in prevalence as pregnancy progressed (Hedman et al., 2002). Many of these symptoms are likely related to increased abdominal mass, fetal movement, and other physiological changes that occur during pregnancy. For example, almost half of pregnant women report symptoms of sleep apnea such as snoring and choking, even though most report they did not snore before pregnancy. Pregnancy is also a risk factor for RLS (Bourjeily et al., 2011; Manconi et al., 2012). RLS symptoms during pregnancy usually occur in the third trimester, when estrogen is at its peak concentration (Berger et al., 2004).

Polysomnographic recordings also reveal changes in sleep architecture across pregnancy. Duration of REM sleep decreases from the first to second trimester (Brunner et al., 1994). The third trimester in particular has the most robust change, with numerous studies showing an increase in wake after sleep onset (WASO) compared to the other trimesters (Karacan et al., 1968; Driver and Shapiro, 1992; Hertz et al., 1992; Brunner et al., 1994; Lee et al., 2000). In the final trimester, women have a longer sleep duration but lower efficiency due to increased WASO and longer sleep latency (Karacan et al., 1968; Lee et al., 2000). REM sleep decreases in the third trimester, while stage 1 (light sleep) of NREM increases and stages 3 and 4 (deep/SWS) decrease (Driver and Shapiro, 1992; Hertz et al., 1992). Spectral analysis of the EEG reveals a progressive decrease in power density during NREM sleep throughout pregnancy, suggesting a decreased depth of sleep in the third trimester (Brunner et al., 1994). In summary, the incidence of reported sleep disturbances increases throughout the course of pregnancy, and this is supported by polysomnographic findings. Most studies have consistently shown increased WASO compared to non-pregnant or postpartum women, likely in part due to disturbances from abdominal enlargement, fetal movement, and lower back pain.

The mother’s hormones continue to fluctuate drastically following parturition, along with increased prevalence of sleep disturbances. In early postpartum, mothers have increased wake time and decreased REM duration, which both return to baseline levels within 2 weeks (Karacan et al., 1968; Driver and Shapiro, 1992; Hertz et al., 1992). Similarly, sleep efficiency decreases in early postpartum but increases 3–5 months later to approximate prepartum levels (Hertz et al., 1992; Lee et al., 2000). Postpartum sleep is also characterized by a longer sleep latency, contributing to the worsened sleep efficiency. However, some measures of sleep quality improve: while deep sleep is drastically reduced in late pregnancy, it is recovered postpartum (Karacan et al., 1968). Most sleep disturbances in this period are related to feeding and caring for the newborn, and could also be impacted by feeding method (Shinkoda et al., 1999; Quillin and Glenn, 2004). It is therefore challenging to distinguish between the effects of hormone changes from the effects of early childrearing on sleep, and both factors are likely involved.

### Menopause

Menopause marks the ceasing of menstruation, after which the ovaries halt production of estrogen and the amount of circulating estradiol and progesterone drops precipitously. Progression through menopause is associated with changes in sleep architecture and an increase in self-reported sleep disruptions. The process of menopause can be separated into three general phases: premenopause, in which menstruation has occurred within the past 3 months; perimenopause, in which menstruation was absent (amenorrhea) for at least 3 months but there has been less than a year of irregular menstrual cycles; postmenopause, in which amenorrhea has occurred for at least a year or the woman has had a complete hysterectomy or bilateral oophorectomy (Young et al., 2003). Due to changes in the menstrual cycle, the menopausal transition is characterized by a decrease in estradiol and increase in FSH, progesterone, and testosterone (Ameratunga et al., 2012).

Subjective sleep measures have been found to worsen with progression through the menopausal transition. The prevalence of reported sleep problems increases from 33–36% in premenopausal women to 61% in postmenopausal women, who have the highest rate of insomnia complaints in the general U.S. population (Kripke et al., 2001). Compared to premenopausal women, postmenopausal women report experiencing more trouble falling and staying asleep, difficulty sleeping, nighttime awakenings, and daytime drowsiness (Ballinger, 1976; Baker et al., 1997; Kravitz et al., 2008). Perimenopausal and postmenopausal women report being less satisfied with their sleep in comparison to premenopausal women (Young et al., 2003). However, in contrast to subjective reports of sleep, objective sleep quality indicators have been found to not decrease during menopause (Ameratunga et al., 2012). Certain objective sleep indicators even improved throughout the transition; compared to pre- and perimenopausal women, postmenopausal women had more SWS, longer sleep duration, and better sleep efficiency (Young et al., 2003). Regardless, numerous studies agree that sleep architecture is altered throughout the different stages of menopause.

Due to the changing hormone profile during menopause, it is reasonable to suggest that sex hormones play a role in sleep disruption during this period. Indeed, a longitudinal study of women progressing through menopause found that decreasing estrogen levels were associated with more frequent awakenings and trouble falling asleep (Kravitz et al., 2008). However, these sleep disturbances could also be age-related, as both men’s and women’s sleep quality decreases in old age. Compared to men, women are more vulnerable to age-related changes in sleep such as decreased reported sleep quality and longer sleep onset latency (Zhang and Wing, 2006; Reyner and Home, 2020). It is likely a combination of age-related changes and hormonal effects that lead to the altered sleep architecture in menopause.

Many women in the menopausal transition use hormone therapy (usually estradiol or a combination of estradiol and progesterone) to alleviate climacteric symptoms such as hot flashes, sweating, headaches, and reported sleep problems (Polo-Kantola et al., 1998, 1999; Hachul et al., 2008). Postmenopausal women using hormones, compared to those who are not, have a lowered risk for disturbed sleep, increased REM sleep, and a shortened onset to sleep latency (Antonijevic et al., 2000; Kravitz et al., 2008). Though the majority of studies indicate that hormone therapy ameliorates subjective sleep quality, exogenously administered estradiol or progesterone does not greatly change sleep architecture (as seen in EEG/EMG recordings) in postmenopausal women (Polo-Kantola et al., 1998, 1999; Montplaisir et al., 2001; Hachul et al., 2008). Regardless, most studies agree that hormone therapy for postmenopausal women can alleviate symptoms and improve reported sleep characteristics.

**FIGURE 1:**
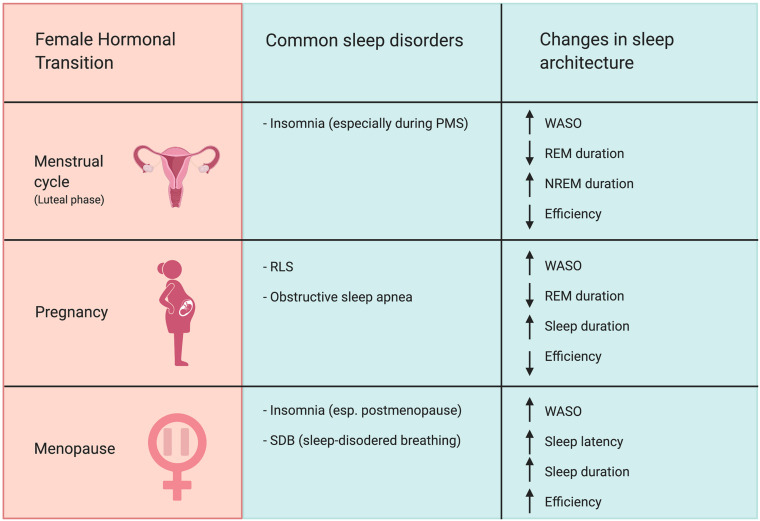
Female hormonal transitions correspond with increased risk for sleep disorders and changes in sleep architecture. The luteal phase of the menstrual cycle, pregnancy, and menopause all increase prevalence of nocturnal awakenings. The luteal phase of the menstrual cycle is associated with increased risk for insomnia. Pregnancy, particularly the third trimester, increases risk of RLS and sleep apnea. The menopausal transition is associated with heightened prevalence of insomnia and SDB, particularly in postmenopause. Abbreviations: WASO, wake after sleep onset; REM, rapid-eye-movement sleep; NREM, non-rapid-eye-movement sleep; PMS, premenstrual syndrome; RLS, restless legs syndrome.

In summary, changes in hormone levels are associated with alterations in sleep architecture (Figure 1). Periods with high progesterone levels, such as the luteal phase of the menstrual cycle, the third trimester of pregnancy, and the menopausal transition, are associated with increased prevalence of self-reported sleep disturbances as well as diagnosis of sleep disorders such as RLS. Periods of change in estradiol levels were also associated with sleep disturbances. Elevated estradiol in the third trimester, hormone therapy, and OCs (although these use synthetic estrogens) changes REM amount and sleep latency. Periods of diminishing estradiol levels, such as during menopause, are characterized by greater risk for insomnia and lowered satisfaction with sleep. Periods of hormonal change in the female reproductive cycle coincide with changes in sleep quality and organization. This clear correlation between hormonal status and sleep strongly suggests that ovarian hormones regulate women’s sleep and may contribute to observed sex differences in sleep. However, in order to causally test this hypothesis, we must look to studies performed in animal models.

## Insights From Animal Models

Animal models are useful tools in biomedical research because they are often easier to biologically manipulate and are more ethical on which to invasively experiment. Rats and mice are the predominant laboratory models of sleep behavior due to the similarities in neurocircuitry they share with humans. Unlike humans, however, rodents have polyphasic sleep, meaning that they have multiple periods of sleep and wake each day. Laboratory rats and mice prefer to sleep in the light phase, but they sleep in periods throughout the entire light–dark cycle. Additionally, while human NREM is subdivided into three to four stages, rodent NREM sleep is subdivided into only two stages. Similar to humans, in rats and other animal models, sleep characteristics differ between males and females. Additionally, sleep architecture changes during hormonal transitions such as the reproductive cycle and hormone replacement.

### Sleep Across the Estrous Cycle

Female rodents, like humans, exhibit changes in both hormones and sleep across their ovulatory cycle. This “estrous cycle” consists of four phases: proestrus, estrus, and diestrus I and II (sometimes referred to as diestrus and metestrus; Levine, 2015). Diestrus I begins with low levels of estrogen and progesterone, which steadily increases in diestrus II. During proestrus, estrogen, LH, and progesterone peak in concentration as ovulation occurs. Behavioral estrus, a period of sexual receptivity (Pfaus et al., 2015), occurs during the active phase (night) of proestrus. The cycle ends with the day of estrus, in which estrogen and progesterone decline rapidly to baseline levels as the cycle restarts. Unlike humans, rodents do not exhibit a prolonged luteal phase with sustained high progesterone.

Female rodents, also like humans, exhibit changes in sleep and locomotor activity across their ovulatory cycle. Sleep in rodents is most altered during proestrus when hormone concentrations change most rapidly. On the day of proestrus, before the peak in estradiol and progesterone levels, rats spend more time sleeping than any other estrous phase (Colvin et al., 1968). Typically, estrogen peaks a few hours before dark phase onset and progesterone peaks early in the dark phase (Smith et al., 1975; Gotlieb et al., 2020). During the night of proestrus, the amount of REM and NREM sleep is markedly reduced in female rats relative to the same time point in other phases of the estrous cycle (Colvin et al., 1968, 1969; Yamaoka, 1978; Kleinlogel, 1983; Fang and Fishbein, 1996; Schwierin et al., 1998; Hadjimarkou et al., 2008; Schwartz and Mong, 2013). Rats had the most brief awakenings in proestrus (Schwierin et al., 1998). Female rodents are most active during the dark phase of proestrus and spend most of their time awake, likely facilitating finding and engaging a sexual partner (Hadjimarkou et al., 2008). After a REM-less night, rodents exhibit REM-rebound during estrus, during which they sleep the most relative to other phases of estrous (Colvin et al., 1968). Schwierin et al. (1998) examined the possibility of sleep homeostasis causing the change in sleep across the estrous cycle. Like other forms of homeostasis, this process drives organisms toward sleep when they digress too far from their wakefulness set point. Sleep homeostasis is regulated by sleep pressure (or sleep drive), which is an organism’s need for sleep at a given time and which increases throughout the day and dissipates during sleep (Borb and Achermann, 1999). Although slow-wave activity and duration of sleep in NREM were changed following sleep deprivation, these effects were identical in estrus and proestrus. Schwierin et al. (1998) concluded that the change in sleep architecture throughout the cycle is not caused by disrupted sleep homeostasis. Overall, female rodents have the lowest amount of sleep during the night of proestrus along with heightened locomotor activity, and this takes place during the peak of estradiol and progesterone concentration. The females then compensate by getting the most sleep the following day, estrus, when the sex hormones return to baseline levels.

### Hormone Manipulation

A major advantage of using animal models is the ability to directly manipulate physiology to probe cause–effect relationships. To experimentally manipulate hormone concentrations in animal models, researchers most frequently remove the gonads (gonadectomy, or ovariectomy for female animals) and may later administer sex hormones to restore physiological concentrations. These studies have been particularly useful to confirm that sex hormones indeed alter sleep duration and sleep architecture.

Notably, removal of sex hormones eliminates sex differences in sleep in rodents. Before gonadectomy, female mice spend more time awake and have less REM sleep in the dark phase than males (Paul et al., 2006). Gonadectomy in rats, mice, and guinea pigs eliminates sex differences in sleep and physical activity and adding back physiological levels of sex hormones restores these differences (Colvin et al., 1969; Yamaoka, 1980; Fang and Fishbein, 1996; Ogawa et al., 2003; Paul et al., 2006; Cusmano et al., 2014). These data strongly indicate that adult sex hormones are critical for sexual dimorphism in sleep behavior.

There is robust evidence that administration of estrogen or of estrogen with progesterone suppresses sleep and promotes wakefulness in females. Estrogen replacement in rats and mice reduces dark phase sleep but has little effect during the light phase (Schwierin et al., 1998; Schwartz and Mong, 2013; Cusmano et al., 2014). Estradiol administration in the dark phase increased wakefulness and decreased duration of REM and NREM sleep, compared to gonadectomized rodents without hormone treatment (Colvin et al., 1968, 1969; Branchey et al., 1971; Paul et al., 2009; Deurveilher et al., 2011; Cusmano et al., 2014). Estradiol therefore clearly exerts an inhibitory effect on REM and NREM sleep. The effect of progesterone alone on sleep is somewhat unclear but is likely to be mild if present at all. Deurveilher et al. (2009) found that progesterone treatment in rats increased REM-sleep latency but decreased the amount of REM sleep. However, Heuser et al. (1967) administered progesterone into the preoptic area of cats, which decreased sleep onset latency and promoted REM sleep. Additionally, Colvin et al. (1969) found that progesterone alone had little or no effect on REM or NREM.

However, when progesterone is administered in conjunction with estrogen, sleep was significantly reduced. Progesterone and estrogen work synergistically to increase time awake and decrease REM and NREM in the dark phase in female rats (Branchey et al., 1971; Deurveilher et al., 2009, 2011). In ovariectomized female guinea pigs, combined estrogen and progesterone treatment reduced REM and NREM sleep (Malven and Sawyer, 1966). Estrogen and progesterone also facilitate recovery from sleep deprivation. In recovery sleep, rats treated with estradiol alone or estradiol with progesterone had more consolidated NREM, longer REM sleep, and fewer awakenings compared to baseline sleep (Deurveilher et al., 2011; Schwartz and Mong, 2013). This suggests that estradiol and progesterone fragment sleep under baseline conditions but help consolidate recovery sleep. Thus, whereas progesterone alone has limited impacts of sleep architecture, it works powerfully in conjunction with estradiol to suppress sleep.

There are considerably fewer studies on the impact of androgens on sleep, but overall it appears the effect of androgens may be sex-specific. The most prevalent androgen in humans and rodent models is testosterone. Testosterone itself is a prohormone that can be either aromatized to estradiol (an estrogen) or reduced to dihydrotestosterone (DHT, a potent androgen), complicating interpretation of studies administering testosterone alone. Cusmano et al. (2014) found that testosterone treatment suppressed REM in female rats but not males or masculinized females. However, treatment with DHT did not affect sleep and wakefulness—indicating that sleep suppression by testosterone was likely due to conversion into estradiol. On the other hand, testosterone or its metabolites may facilitate sleep in males. Male mice treated with testosterone experienced an increase in NREM sleep and a decrease in wake amount compared to vehicle-treated males (Paul et al., 2009). Testosterone has also been associated with sleep recovery following sleep disruption, as administration of testosterone following sleep deprivation promoted NREM recovery sleep in male mice (Paul et al., 2009).

### Organizational Effects of Sex Hormones on Sleep

The aforementioned work addresses only the impact of removing and replacing gonadal steroids in adult animals. However, there is significant evidence that exposure to sex hormones early in life can shape lifelong sleep characteristics. According to the organizational-activational model of sexual differentiation, gonadal hormones can either guide neural development (“organization”) or tune activity within circuits (“activation”), largely dependent on the age of the organism (Phoenix et al., 1959; Arnold, 2009). Gonadal hormone exposure perinatally organizes tissues that regulate masculine or feminine behaviors and modulates sensitivity to sex hormones in the future. Classically, Phoenix et al. (1959) found that prenatal testosterone treatment in female guinea pigs reduced lordosis behavior and increased mounting behavior in adult animals, permanently altering their behavior. Blocking hormone exposure prenatally in males also results in increased feminized behavior and decreased masculine behavior in adult animals (Thornton et al., 1991). In adulthood, gonadal hormones act on existing brain circuits to promote expression of sexually differentiated behavior (as seen in the data discussed above).

Indeed, gonadal hormones exert an organizational effect on adult sleep behavior. The masculinized brain has been found to be less sensitive to the effects of sex hormones on sleep. Male rats castrated neonatally—before the brain has completed masculinization—exhibited reduced REM and NREM duration after estradiol and progesterone treatment (Branchey et al., 1973), more similar to female rats. However, female rats that were exposed to masculinizing sex hormones prenatally and male rats castrated in adulthood show little to no change in sleep patterns following hormonal treatment (Branchey et al., 1973; Yamaoka, 1980; Cusmano et al., 2014). These data indicate that early life exposure to sex steroids organizes the brain such that adult males are “protected” from estradiol’s effects on arousal and sleep.

## Direct Impact of Ovarian Hormones on Neural Sleep Circuits

Clearly, ovarian hormones regulate female sleep. However, the neurobiological substrates mediating this effect remain elusive. Sleep and wakefulness are regulated by numerous interconnected neural loci that are often conceptualized as either sleep-promoting or wake-promoting. Major wake-promoting brain regions include cholinergic neurons from the laterodorsal tegmental (LDT) nuclei and pedunculopontine tegmental (PPT) nuclei, histaminergic neurons from the tuberomammillary nucleus (TMN), serotonergic raphe nuclei that all project throughout the forebrain (Herkenham, 1980; Steininger et al., 1999), norepinephrinergic neurons of the locus coeruleus (LC), and hypocretin-expressing neurons of the lateral hypothalamic area (Peyron et al., 1998; Sakurai et al., 1998; Jennings and de Lecea, 2019). Perhaps the best studied sleep-promoting brain region is the ventrolateral preoptic (VLPO) area, which contains sleep-active GABAergic and galaninergic neurons (Sherin et al., 1998; Saper and Fuller, 2017), although additional sleep-promoting neural populations continue to be identified (Xu et al., 2015; Liu et al., 2018; Weber et al., 2018).

Female sex hormones impact a wide array of neural mechanisms implicated in sleep. There are estrogen and progesterone receptors in numerous sleep- and arousal-regulating brain regions such as the preoptic area, SCN, LC, and other hypothalamic nuclei (Shughrue et al., 1997; Curran-Rauhut and Petersen, 2002). As we will discuss below, estradiol in particular directly and indirectly influences the activation of neurons in these regions, likely with consequences on sleep/wake functioning that have not yet been fully explored.

### Ventrolateral Preoptic Area

The VLPO area is one of the major brain regions associated with sleep promotion. VLPO neurons increase their firing rate during sleep compared to waking (Szymusiak et al., 1998). Estradiol influences the activation of these sleep-promoting neurons. In ovariectomized female rats, estradiol decreased VLPO activation (as measured by c-Fos expression) under baseline conditions (Deurveilher et al., 2008; Hadjimarkou et al., 2008).

Estradiol may influence the activation of VLPO neurons by regulating lipocalin-type prostaglandin D synthase (L-PGDS) and adenosine levels. L-PGDS is an enzyme that catalyzes prostaglandin H_2_ (PGH_2_) to prostaglandin D_2_ (PGD_2_), which induces sleep when applied to the preoptic area (Ueno et al., 1982). Inhibiting L-PGDS catalytic activity in the VLPO reduces sleep and increases wake time (Mong et al., 2003b; Qu et al., 2006). Similarly, administration of estradiol to the VLPO in rats decreases L-PGDS transcript levels by half (Mong et al., 2003a,b; Ribeiro et al., 2009). Consequently, PGD_2_ expression is lowered, causing sleep problems. Hadjimarkou et al. (2008) found that treating female rats with estradiol reduced the female’s concentrations of L-PGDS protein to the level of male rats. They also found that blocking estradiol from acting on the preoptic nucleus prevented estrogen-mediated suppression of sleep. Estradiol regulates L-PGDS promoter activity through estrogen receptors, especially ERα, which has been shown to promote L-PGDS activity (Lightfoot and Gym, 2008; Devidze et al., 2010).

Estradiol may also regulate sleep by acting on adenosine receptors, which mediate the somnogenic effects of PGD_2_. Adenosine concentration in the brain steadily increases during the waking period and decreases during sleep, leading some to suggest a role in the biological basis of sleep pressure (Porkka-Heiskanen et al., 1997). Adenosine inhibits forebrain and mesopontine cholinergic neurons involved in arousal, allowing it to regulate wake and sleep (Portas et al., 1997). When administered to the preoptic area, estradiol decreased adenosine A_2__*A*_ receptor mRNA levels and increased running wheel activity in mice (Ribeiro et al., 2009). Huang et al. (2007) proposed that PGD_2_ indirectly excites VLPO neurons by stimulating adenosine release into the VLPO. Therefore, when estradiol decreases the amount of adenosine A_2__*A*_ receptors in the VLPO, not as much adenosine is released, which decreases the activation of VLPO neurons. In summary, estradiol decreases L-PGDS and A_2__*A*_ receptor transcription levels in the VLPO, increasing arousal.

### Lateral Hypothalamus

The lateral hypothalamus has also been implicated in sleep/wake regulation. The lateral hypothalamus is populated with neurons which express the neuropeptide hypocretin (Hcrt, also known as orexin), a neuroexcitatory peptide associated with a broad range of functions in sleep, arousal, and appetite, among others (de Lecea et al., 1998; Sakurai et al., 1998; Jennings and de Lecea, 2019). Hcrt1 and 2 (also called Orexin A and B) are released from neurons in the lateral hypothalamus which project widely in the brain, including to structures involved in sleep–wake such as the preoptic area, LC, raphe nuclei, and TMN (de Lecea et al., 1998; Peyron et al., 1998; Chemelli et al., 1999).

The hypocretins promote and stabilize wakefulness. Disruption of hypocretin neurotransmission causes narcolepsy in mice, dogs, and humans (Chemelli et al., 1999; Lin et al., 1999; Nishino et al., 2000). The absence of Hcrt impairs the maintenance of the waking state, causing narcoleptic symptoms such as sleep attacks. Hcrt neurons fire more during waking and REM than SWS, suggesting that Hcrt plays a role in activating the ascending arousal system (Takahashi et al., 2008). Furthermore, photostimulation of Hcrt neurons in the lateral hypothalamus promoted sleep-to-wake transitions and decreased sleep-to wake latency, regardless of the time of day (Adamantidis et al., 2007; Carter et al., 2009). Therefore, the neuropeptide hypocretin is strongly associated with promoting and stabilizing arousal. Hcrt-containing neurons innervate components of the ascending arousal system and the VLPO, suggesting that hypocretin could regulate both sleep and wake (Saper et al., 2001).

Hypocretin expression is sexually differentiated, likely due to regulation by reproductive hormones. Preprohypocretin (a precursor for Hcrt) mRNA levels are greater in the hypothalamus of female rats than males (Jöhren et al., 2002). Consequently, female rats have greater concentrations of Hcrt1 compared to males (Taheri et al., 1999). The Hcrt system is sensitive to fluctuations in ovarian hormones. Estradiol increases c-Fos expression in Hcrt-containing hypothalamic neurons, suggesting that estradiol could promote wakefulness by increasing activation of Hcrt neurons (Deurveilher et al., 2008). Production of Hcrt and its receptors changed during the course of the rodent reproductive cycle. Hcrt1 and 2 concentration as well as HcrtR1 protein expression peaked in the hypothalamus during the day of proestrus, which coincides with the surge of progesterone and estradiol (Porkka-Heiskanen et al., 2004; Silveyra et al., 2007). Ovariectomized female rats, on the other hand, exhibited a decrease in HcrtR1 expression in the hypothalamus, and estradiol replacement restored Hcrt expression to the high levels of normally cycling rats (Silveyra et al., 2007, 2009). Conversely, examining the Hcrt regulation of sex steroid production reveals that hormonal status may moderate the impact of Hcrt signaling. Hcrt stimulated release of LH when administered to ovariectomized female rats, but only when they had been primed by estrogen and progesterone. Without female reproductive hormone priming, Hcrt inhibited LH secretion (Pu et al., 1998). This suggests that estradiol and progesterone somehow contextually flip hypocretin’s influence on gonadotropin release and raises the possibility of sex hormones similarly moderating the impact of Hcrt on other neural systems, such as sleep/wake. Androgens may also regulate Hcrt signaling, at least in males. In male rats, gonadectomy decreased HcrtR1 expression in the anterior hypothalamus, and both testosterone and DHT restored receptor expression (Silveyra et al., 2009). Since DHT is a non-aromatizable androgen, these data suggest that androgen receptor signaling can regulate HcrtR expression, potentially impacting sleep and wake drives. Overall, sex steroids, particularly ovarian sex hormones, have been implicated in enhancing the activity of hypocretin neurons. Ovarian hormones may therefore enhance hypocretin’s ability to strengthen the arousal drive and prevent inappropriate switching to the sleep state.

### Tuberomammillary Nucleus

Histaminergic neurons in the TMN of the posterior hypothalamus are another wake-promoting population which has been shown to be regulated by ovarian hormones. These histaminergic neurons fire specifically during wakefulness and stop firing during deep NREM sleep and REM sleep (Vanni-Mercier et al., 2003). Knockout mice without histamine had decreased sleep latency, shorter wake episodes, and more REM sleep (Parmentier et al., 2002). Inhibiting histamine transmission increases NREM sleep and decreases wakefulness, while enhancing histamine transmission has the opposite effect (Lin et al., 1988).

Ovarian hormones influence the activation and activity of histamine-related molecules. Estradiol treatment increases c-Fos expression in histaminergic TMN neurons compared to the vehicle (Deurveilher et al., 2008). Histamine receptor binding sites are sexually dimorphic in rats, and ovariectomy removed sex differences whereas estradiol treatment restored them (Seltzer and Donoso, 1989; Ghi et al., 1991). However, progesterone blocked estradiol-mediated recovery of sex differences in binding sites, suggesting that both hormones work to regulate histamine transmission. These hormone-driven changes in TMN histaminergic signaling could likely influence the role of TMN neurons in promoting wakefulness.

### Locus Coeruleus

The LC is located in the pons of the brainstem, and it is the primary site of norepinephrine (NE) production in the central nervous system. NE is released upon activation of the sympathetic nervous system to increase heart and breathing rate, and slow digestion to respond to the stressor. Sympathetic nervous system activation is also the quicker, more immediate component of the stress response system (Russell and Lightman, 2019). Thus, noradrenergic neurons in the LC regulate arousal responses. The LC is sexually dimorphic: female rats compared to males have greater total LC volume and more neurons containing dopamine-beta-hydroxylase (DBH), an enzyme which catalyzes dopamine to NE (Luque et al., 1992). This suggests that females can synthesize and release more NE, increasing their arousal. Ovarian hormones also influence activity in the LC. Estradiol increased c-Fos expression within LC cells in sleep-deprived rats compared to non-deprived controls, suggesting that estradiol potentiates LC neuron activation (Deurveilher et al., 2008). Estradiol and progesterone together increase extracellular NE levels in the LC, likely due to estradiol’s influence on enzymes related to NE (Vathy and Etgen, 1988). Estradiol increases levels of tyrosine hydroxylase (TH), the rate-limiting enzyme in the synthesis of NE, and also increases DBH concentration (Serova et al., 2002). It has been proposed that estradiol’s enhancing effect on NE activity in the LC could make females more vulnerable to hyperarousal (Bangasser et al., 2016, 2019). This state of heightened arousal could thus promote wakefulness and inhibit sleep in females, leading to reports of disrupted sleep.

### Dorsal Raphe Nucleus

Serotonergic neurons in the dorsal raphe nucleus (DRN) are involved in the regulation of wake and REM (Monti, 2010). Suppressing serotonergic activity in the DRN has been shown to increase REM sleep (Portas et al., 1996). Estradiol regulates several serotonin [5-hydroxytryptamine (5-HT)] receptor subtypes. The 5-HT_1__*A*_ autoreceptor detects extracellular serotonin and then inhibits serotonin neural activity in a negative feedback loop (Bethea et al., 2002). Systemic administration of estradiol and progesterone decreases 5-HT_1__*A*_ autoreceptor binding sites in the DRN, increasing serotonin transmission (Lu and Bethea, 2002). Similarly, activating the 5-HT_2__*A*_ receptor reduces REM (Monti and Jantos, 2006) while blocking the receptor increases NREM and decreases wake (Popa et al., 2005). Estradiol treatment increases the expression of 5-HT_2__*A*_ receptors in the DRN, increasing 5-HT concentration (Sumner and Fink, 1993; Sumner et al., 1999). Considered together, ovarian hormones downregulate 5-HT_1__*A*_ autoreceptors while upregulating 5-HT_2__*A*_ receptors, which increases transmission of serotonin. This could potentially explain why female rats exhibit greater DRN serotonergic activity than males (Domínguez et al., 2003). Furthermore, neonatal treatment with gonadal sex hormones altered serotonergic activity in the DRN in adults, suggesting that organizational effects of these steroids could also be involved. Overall, ovarian hormones increase serotonin transmission in the DRN which likely strengthens arousal, promoting wake and suppressing NREM and REM.

### Basal Forebrain Cholinergic Neurons

The basal forebrain is another region implicated in promoting wakefulness. Basal forebrain cholinergic neurons (BFCN) promote arousal and increase wake and REM when stimulated (Xu et al., 2015). Like other components of the arousal circuit, BFCN are sexually dimorphic. Young male rats have larger BFCN than young females (Veng et al., 2003). Ovarian hormones modify morphology and transmission in BFCN. Estradiol exerts a protective effect on cholinergic neurons by enhancing neurotrophin receptor expression (Henderson et al., 1996; Ábrahám et al., 2009). Alzheimer’s disease is characterized by the degeneration of BFCN, so estradiol’s ameliorative effect on BFCN during neurodegeneration makes it a potential candidate for treatment (Henderson et al., 1996). Estradiol also increases cell soma size in this region and increases choline acetyltransferase (ChAT) activity—an enzyme that synthesizes acetylcholine—in projection areas of BFCN. Considered together, ovarian hormones facilitate and support cholinergic transmission in the basal forebrain, and therefore promote wakefulness.

### Ventral Tegmental Area

Dopaminergic neurons in the ventral tegmental area (VTA) are well known for their role in motivation, learning, and reward (Wise, 2004; Bromberg-Martin et al., 2010). However, this region has also been implicated in promoting arousal. Stimulation of dopaminergic VTA neurons initiated and maintained wakefulness, while inhibition suppressed wakefulness and promoted sleep-nesting behavior (Eban-Rothschild et al., 2016). While the VTA is not noticeably structurally dimorphic between males and females (Chung et al., 2017), there are sex differences in dopaminergic transmission. Most of the VTA’s effects on promoting wake are mediated through major projections to the nucleus accumbens (NAc). VTA dopaminergic neuron firing rate and dopamine release into the NAc are higher during vaginal estrus than other phases of the reproductive cycle (Zhang et al., 2008), although this is counterintuitive because rats sleep the most in estrus. Cocaine administration produced a greater inhibition of the firing of VTA dopaminergic neurons during proestrus than estrus. This inhibitory effect was blocked by ovariectomy and restored by estradiol, which increased dopaminergic neuron activity. During estrus, cocaine was mediated by estradiol to have an increased affinity to bind to DAT (dopamine transporter) and inhibit the uptake of dopamine (DA), increasing DA concentration (Calipari et al., 2017). Overall, DA transmission in the VTA varies throughout the estrous cycle and estradiol can modulate cocaine’s impact on dopamine release. DA neurotransmission has been associated with sleep/wake states, and sleep modifies the potency of cocaine (Alonso et al., 2020). Considering the VTA-NAc circuit promotes arousal, this indicates that ovarian hormones could potentiate dopaminergic transmission in the VTA to promote wakefulness (although this remains to be tested).

### Glia

Glial cells roughly equal neurons in number (von Bartheld et al., 2016), and their myriad of significant roles distinguish them as a new frontier of neuroscience. Glial cells have been implicated in many roles in the nervous system, including regulation of sleep (Frank, 2019). Astrocytes support neurons and have been implicated in sleep homeostasis. Impairing gliotransmission in astrocytes decreases slow-wave activity, an indicator of sleep pressure (Halassa et al., 2009). Astrocytes are the most numerous cells in the human brain and are located throughout the entire central nervous system, including regions associated with sleep such as the lateral hypothalamus. One mechanism through which astrocytes regulate sleep is through secretion of somnogens. Cultured astrocytes secrete interleukin-1 (IL-1), which increases slow-wave activity (Tobler et al., 1984). Sex differences in glia have been reported, and these non-neural cells are also regulated by gonadal hormones. Bollinger et al. (2019) found sex-specific effects of stress on glia in the medial prefrontal cortex (mPFC) that were hormone-dependent. Stress increased microglial density in the mPFC of female rats, while stressed males had a greater astrocyte area. Gonadectomy increased microglial area in males, which was prevented by testosterone treatment. In females, ovariectomy blocked stress effects on microglia, which were restored by estradiol. Sex hormones differentially modify glia morphology in the context of stress, and perhaps this could occur in other neural responses such as the sleep/wake drive. Glial cells are regulated by many nuclear receptors such as ERβ, the dominant estrogen receptor in microglia (Saijo et al., 2013). Estrogen also acts as a neuroprotectant for glial cells (Morale et al., 2006; Arevalo et al., 2010) because ERβ represses inflammatory responses of microglia and astrocytes (Saijo et al., 2011). Estrogen receptors mediate glial inflammatory responses, suggesting that ovarian hormones mediate glial activity. There is a knowledge gap of how hormone-mediated changes to glial morphology and activity relate to sleep, but changes to glia could impact other neural regions associated with sleep and wake.

### Female Sex Hormones Promote Wakefulness and Suppress Sleep

**FIGURE 2:**
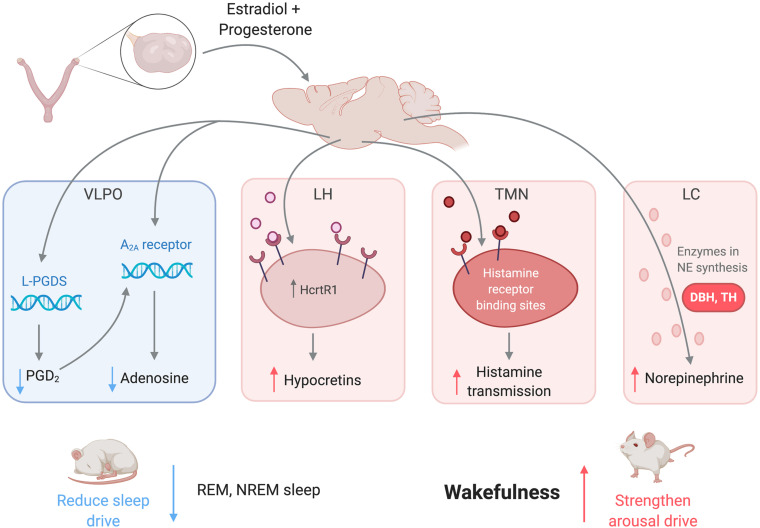
Female sex hormones act on neural regions associated with sleep and wakefulness to promote arousal and suppress sleep. Estradiol and progesterone suppress neural transmission of somnogens in the VLPO, weakening the sleep drive. The hormones also increase transmission of arousal-promoting substances in the LH, TMN, LC, as well as other regions not pictured to strengthen the arousal drive. Abbreviations: VLPO, ventrolateral preoptic nucleus; L-PGDS, lipocalin-type prostaglandin synthase; PGD_2_, prostaglandin H_2_; A_2__*A*_, adenosine 2A receptor; LC, locus coeruleus; NE, norepinephrine; TMN, tuberomammillary nucleus; LH, lateral hypothalamus; HcrtR1, hypocretin receptor 1; REM, rapid-eye movement sleep; NREM; non-rapid-eye movement sleep.

In summary, female sex hormones act on numerous brain regions associated with sleep and arousal to promote wakefulness and consolidate sleep periods (Figure 2). Estradiol downregulates PGD_2_, a somnogen, in the VLPO to reduce sleep drive (Mong et al., 2003a,b). Both estradiol and progesterone regulate histamine transmission, which could mediate the arousal-enhancing role of histaminergic neurons in the TMN (Deurveilher et al., 2008). Estradiol could also promote wakefulness by upregulating NE activity in the LC (Vathy and Etgen, 1988), serotonergic activity in the DRN (Lu and Bethea, 2002), dopaminergic activity in the VTA-NAc circuit (Zhang et al., 2008), hypocretinergic activity in the LH (Silveyra et al., 2007), and/or cholinergic activity in the BF (Henderson et al., 1996). Glia, which have been implicated in sleep homeostasis, are also regulated by gonadal steroids and potentially impact many regions in sleep/wake circuits (Morale et al., 2006). All of these actions by ovarian hormones are consistent with the hypothesis that ovarian hormones act widely throughout the brain to promote wake and suppress sleep. Notably, although sexual dimorphism and sex hormone regulation of these regions are well established, the extent to which any given region may mediate the hormonal regulation of sleep remains to be experimentally tested. Additionally, most of the regions described contribute to the arousal system, so there is more room for exploration among sleep-promoting regions.

## The Impact of Hormones on Sleep-Related Systems

### Nesting and Locomotor Behaviors

There have also been reports of sex differences and hormone treatment effects on systems related to or supportive of sleep that could influence sleep and wakefulness regulation. Nesting behavior, which includes the actions involved in making a nest, is hormonally controlled and is usually studied in the context of parental behavior (Lisk et al., 1969). Nest building also supports sleep behavior (Eban-Rothschild et al., 2016), and it is currently unknown how sex hormones regulate the impact of nest building on sleep in non-parental animals. Hormone treatment also impacts locomotor activity. In female rats, levels of ovarian hormones and estrogen receptors influenced voluntary locomotor activity (Gorzek et al., 2007). Ovariectomy and estrogen deficiency both decreased wheel running, which was restored by estrogen treatment (Ogawa et al., 2003; Vyazovskiy et al., 2006; Gorzek et al., 2007; Ribeiro et al., 2009). Estradiol increases locomotor activity, which may increase general arousal and limit the time available to sleep.

### Circadian Rhythms

One of the most significant sleep-related systems not yet discussed is circadian biology. The suprachiasmatic nucleus (SCN) in the anterior hypothalamus is the central pacemaker of the circadian system, regulating the timing of sleep and physical activity (Rusak and Zucker, 1979). The circadian system synchronizes day and night (light and dark) with an organism’s internal processes to create roughly 24-h circadian rhythm. Circadian rhythms shift in response to external factors such as light, exercise, and feeding (Stephan, 2002; Abbott et al., 2015). As part of synchronizing physiologic and behavioral processes to the environment, the circadian system consolidates sleep into longer periods at the appropriate time of day as opposed to erratic bouts. Circadian rhythms are primarily orchestrated by the SCN, which if lesioned causes animals to sleep in unconsolidated periods, independent of the light and dark cycle (Stephan and Zucker, 1972; Mouret et al., 1978).

Ovarian hormones have been shown to alter circadian rhythmicity, which directly impacts sleep–wake activity. Rodents who are gonadectomized or otherwise estradiol deficient have fragmented sleep and are less entrained to the light phase, but estradiol treatment restored the circadian timing of sleep (Colvin et al., 1969; Vyazovskiy et al., 2006). Therefore, estradiol has been proposed to consolidate circadian sleep–wake rhythms in female rats (Schwartz and Mong, 2013). Animal models also exhibit changes in measures of circadian activity throughout the female reproductive cycle. Female rats and hamsters showed the most change during proestrus and estrus (the days of the greatest change in estrogen and progesterone levels), during which the animals exhibited shortened periods and advanced phases of physical activity (Morin et al., 1977; Albers et al., 1981).

The SCN is a sexually dimorphic structure in many aspects, although the functional consequences of these differences are not completely understood. The volume of the SCN is larger in male rats than females (Gorski et al., 1978; Robinson et al., 1986). The SCN of male rats also has more axo-spine synapses, postsynaptic density material, and asymmetrical synapses (Güldner, 1982, 1984). These structural and synaptic differences could indicate differences in SCN activity between males and females. There is also evidence for hormonal regulation of clock genes in the SCN. Estradiol treatment increased mRNA levels of *Cry2*, a clock gene, in the SCN while ovariectomized female rats had lowered gene expression (Nakamura et al., 2001). Many questions remain regarding the direct impact of ovarian hormones on SCN activity, although preliminary evidence suggests that estrogen impacts circadian rhythms by acting through the SCN. Estrogen receptor mRNA expression in the SCN of rats fluctuates according to a diurnal pattern, suggesting a correlation between estrogen signaling and circadian rhythms (Wilson et al., 2002). Future studies are needed to better understand how ovarian hormones may impact SCN functioning with consequences for circadian rhythms, including sleep.

### Stress

The link between stress and sleep is robust, and it is generally accepted that stress impairs sleep (Friedman et al., 1995; Bastien et al., 2004; Kalmbach et al., 2018). In addition to norepinephrinergic activity in the LC, other sex-differentiated stress responses can contribute to changes in sleep. Like the HPG axis, the hypothalamic-pituitary-adrenal (HPA) axis includes a cascade of neural and hormonal signals, triggered by circadian and environmental indicators. Distinct from the sympathetic nervous system, the HPA axis is the slower, secondary component of the stress response. Within the HPA, the periventricular nucleus (PVN) of the hypothalamus releases corticotropin releasing hormone/factor (CRH/CRF) (Vale et al., 1981). This triggers the anterior pituitary to release adrenocorticotropic hormone (ACTH), causing the adrenal cortex to release corticosteroids (CORT) in preparation to respond to the stressor. Circulating CORT then acts on the hypothalamus and pituitary in a negative feedback loop to regulate hormone secretion. Like other stress systems, the HPA has been correlated with wakefulness. Sleep deprivation increases HPA activity, and optogenetic activation of CRF neurons within the HPA promotes wakefulness while chemogenetic inhibition decreases wake time (Minkel et al., 2014; Li et al., 2020; Ono et al., 2020). The HPA axis has also been correlated with circadian rhythmicity, suggesting the possibility that HPA activity could influence the circadian system to regulate sleep (Kalsbeek et al., 1995; Tousson and Meissl, 2004). There is a sex difference in stress responses within the HPA, with females exhibiting a stronger response than males (Gaskin and Kitay, 1970; Brett et al., 1983; Handa et al., 1994; Handa and McGivern, 2016). There is also a female-biased sex difference in both baseline and stress-induced levels of ACTH expression in the PVN (Handa et al., 1994; Iwasaki-Sekino et al., 2009). Altered HPA functioning has been associated with generalized anxiety disorder (GAD) and other anxiety disorders (Mantella et al., 2008; Dieleman et al., 2015), which may be related to women’s greater risk for anxiety disorders (Kessler et al., 2005; Bekker and Van Mens-Verhulst, 2007). Gonadal hormones regulate the sexually differentiated functioning of the HPA. Estrogen treatment potentiates ACTH and CORT secretion in response to stress, while testosterone attenuates the response (Burgess and Handa, 1992; Handa et al., 1994). Given the link between the HPA axis and wakefulness, sex differences in HPA functioning could contribute to the sex difference in sleep. The greater reactivity of the HPA axis in response to stress could increase general arousal in females, strengthening the wake drive.

## Discussion

**FIGURE 3:**
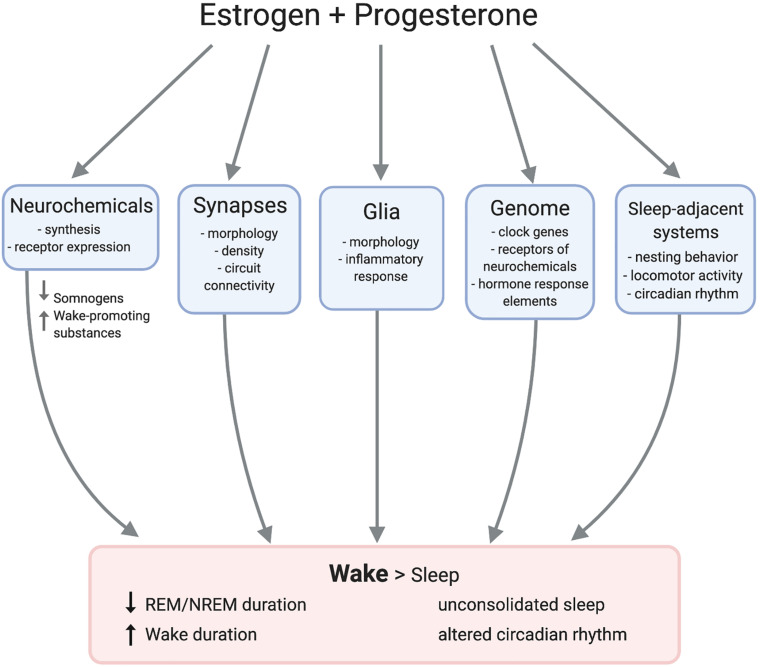
Female sex hormones act on adjacent mechanisms to promote wake over sleep. Estradiol and progesterone modulate neurochemical transmission and morphology of synapses and glia. Hormone response elements such as estrogen response elements (EREs) directly impact gene expression, possibly regulating clock genes. Estradiol and progesterone also impact nest building and circadian rhythms, resulting in longer duration of wake than sleep.

Multiple converging lines of evidence substantiate that ovarian hormones can regulate sleep in both humans and animal models. Studies have repeatedly demonstrated strong correlations between natural hormonal fluctuations and sleep across species. Experimental manipulations support this model and indicate that estrogen signaling is by far the most impactful on sleep. However, the exact neurobiological mechanisms mediating the hormonal control of sleep remain elusive. Ovarian hormones impact the physiology of numerous sleep- and wake-regulating neural loci such that wake is promoted over sleep (Figure 3). This array of neurobiological changes likely contributes to the observed sexual dimorphism in sleep behavior, and potentially to women’s elevated risk of sleep disruption across the lifespan.

There is clearly a significant knowledge gap in our mechanistic understanding of how ovarian hormones impact the brain to regulate sleep. For example, estradiol modifies histamine binding sites in the TMN, but there are likely additional pathways by which estradiol could impact transmission of histamine or other compounds associated with wakefulness in the TMN. It is known that estrogen alters circadian rhythmicity, but the impact of estrogenic signaling on molecular pathways and neuronal functioning in the SCN is unclear. It is also evident that ovarian hormones regulate LC structure and neurochemistry. However, studies on estrogen in the LC have primarily considered the hormone’s impact on hyperarousal in the context of stress, so further research is needed to test whether estrogenic regulation of the LC affects sleep. It is also unknown whether sex hormones regulate sleep through actions on glial cells, which comprise about half of the brain and interact with neural transmission.

Ovarian hormones not only impact physiology within the aforementioned regions, but they may also regulate information flow across regions. As has been proposed for social behavior circuits (Newman, 1999; Goodson and Kabelik, 2009), sex hormones could regulate the information weighting and functional connectivity within sleep/wake circuits to produce alterations in sleep behavior. Estrogen has been shown to have region-specific effects on inter-region connectivity. For example, synaptic plasticity and functional connectivity in the ventromedial hypothalamus to anteroventral PVN pathway change throughout the estrous cycle with direct consequences to female reproductive behavior (Inoue et al., 2019). At the electrophysiological level, estradiol administration decreased excitability of medial amygdala afferents from the medial preoptic area but not those from the lateral septum, indicating that hormonal regulation of computational inputs can be region-specific (Yoshida et al., 1994). Notably, these effects have primarily been studied in the context of hormonally-regulated social behavior circuits. However, we argue that sleep is also a hormonally-regulated behavior, and thus is potentially regulated by similar mechanisms. Further research should elucidate the role of ovarian hormones in modulating the pattern—not just the activation in isolation—of neural networks to guide sleep and wake behavior.

Our understanding of the mechanisms of sexual dimorphism within the sleep/wake circuits is limited, although rodent models indicate that gonadal steroids exert an organizational effect on this circuitry early in life. Another less explored potential contributor to sexual dimorphism is the impact of sex chromosomes, which differentiate neurons into XX or XY cells based on the biological sex of the organism (Arnold, 2004). Sleep pressure following sleep deprivation increases more rapidly in women than men, and sex chromosomes have been implicated in this process (Paul et al., 2008; Ehlen et al., 2013). Ehlen and colleagues tested the impact of sex chromosomes on sleep using mice from the four core genotypes mice model, in which the sex chromosome complement (XX, XY) is independent of the mouse’s gonadal sex (male or female). The four genotypes are XX females, XX males, XY females, and XY males. During sleep recovery following sleep deprivation, XY female mice slept more than XX females (Ehlen et al., 2013). Further studies directly testing the impact of sex chromosomes on sleep neurobiology would complement work on sex hormones and sleep.

The ultimate adaptive benefit of sex differences in sleep is unknown (if one ever existed at all), but it is clearly maladaptive in modern society. Ovarian hormones regulate the neurocircuitry related to sleep such that women are biologically predisposed for disrupted sleep. This is a public health concern because sleep is crucial for optimal health and well-being, and its disruption leads to dire consequences. For example, sleep deprivation increases risk for cardiovascular disease, hypertension, and Alzheimer’s disease (Meng et al., 2013; Vgontzas et al., 2013; Smith and Mong, 2019). Sleep disturbances are also associated with cognitive deficits such as reduced memory consolidation and inattentiveness, as well as psychiatric problems such as substance abuse (Drummond and Brown, 2001; Johnson et al., 2006; Gujar et al., 2010). Therefore, women’s increased risk of sleep problems could predispose women for a higher risk of disease and cognitive deficits. Further research is required in this area to understand and address the sleep disturbances that disproportionately burden half of the world’s population.

In spite of the clear necessity to understand and improve women’s health, many unanswered questions remain. One reason why this knowledge gap has persisted is because historically most sleep studies have used only male subjects. This is a broad issue across biomedical sciences. A meta-analysis found that neuroscience studies using only male animals outnumbered female-only studies by a ratio of 5.5:1, and few studies in this field used both male and female subjects (Beery and Zucker, 2011). The sex bias in animal models is significant because as of 2009, 85% of neuroscience studies used rodents as subjects. Researchers often defend their choice to only include males by claiming that it is challenging to control for the variation in length of reproductive cycles between females. However, studies testing pain receptivity, gene expression, and other neuroscience-related traits found female mice tested at different points in their cycle to be no more variable than male mice (Prendergast et al., 2014; Itoh and Arnold, 2015; Becker et al., 2016). In fact, male mice exhibited a significantly greater trait variability than females in these studies. Women and female animals have long been excluded from sleep studies, leading to a knowledge gap in how mechanistic sleep circuitry differs between males and females and how ovarian hormones might impact these circuits. Neuroscience and sleep research are improving in this aspect, but experimenters should continue to prioritize studies with sex as a biological variable in order to understand how female physiology interacts with sleep biology.

Better inclusion of women in pharmaceutical clinical evaluation could pave the way for sleep treatment tailored for women. Most pharmaceutical treatments have been designed primarily according to men and male physiology due to the longstanding sex bias in biomedical research, including sleep research. This male bias in basic research has led to deleterious consequences for women even confining our discussion to sleep treatments. For example, in 2013, the FDA reduced the recommended dose of Ambien (zolpidem), a popular sleep aid, for women due to sex differences in drug metabolism leading women to experience high dosages exceeding safe levels (FDA Drug Safety Communication, 2014). Administration of olanzapine, another sleep aid, increased NREM in women but decreased NREM in men (Giménez et al., 2011). Given the sex differences in the effects of pharmaceutical pharmacological treatment on sleep, future research should determine optimal treatment and doses for sleep problems in women.

Finally, we reiterate that the data on “women” in this review come from and apply to cis-women. This is because sex and gender minorities have been and continue to be understudied in biomedical research, including sleep research. Future research on these populations could improve health outcomes for these historically underserved populations. In particular, trans-men and women receiving gender-affirming hormone treatment represent a uniquely positioned population for studying the organizational versus activational effects of sex hormones on sleep in humans. Women in general have been understudied in biomedical research, and we encourage that more research and resources be applied to benefit additionally marginalized groups.

## Conclusion

Women have significantly more sleep problems than men, likely in part due to female sex hormones. Estrogen and progesterone change sleep architecture, and ovarian hormones impact neural transmission in areas related to sleep/wake regulation. However, further research can be done in this field. Elucidating the mechanisms behind sex differences in sleep could help not only to reduce sleep disturbances in women, but also to promote sleep health and well-being.

## Author Contributions

AD wrote the manuscript and designed the figures. All authors discussed and edited the manuscript.

## Conflict of Interest

The authors declare that the research was conducted in the absence of any commercial or financial relationships that could be construed as a potential conflict of interest.
